# Metabolic Memory Phenomenon and Accumulation of Peroxynitrite in Retinal Capillaries

**DOI:** 10.1155/2007/21976

**Published:** 2007-06-25

**Authors:** Renu A. Kowluru, Mamta Kanwar, Alexander Kennedy

**Affiliations:** Kresge Eye Institute, Wayne State University, Detroit, MI 48201, USA

## Abstract

*Aim*. Diabetic retinopathy resists reversal after good glycemic control (GC) is reinitiated, and preexisting damage at the time of intervention is considered as the major factor in determining the outcome of the GC. This study is to investigate the role of peroxynitrite accumulation in the retinal capillaries in the failure of retinopathy to reverse after reestablishment of GC, and to determine the effect of this reversal on the activity of the enzyme responsible for scavenging mitochondrial superoxide, MnSOD. *Methods*. In streptozotocin-diabetic rats, 6 months of poor glycemic control (PC, glycated hemoglobin, GHb > 12.0%) was followed by 6 additional months of GC (GHb about 6%). The trypsin-digested retinal microvessels were prepared for immunostaining of nitrotyrosine (a measure of peroxynitrite) and for counting the number of acellular capillaries (a measure of histopathology). The retina from the other eye was used to quantify nitrotyrosine concentration, MnSOD activity and the total antioxidant capacity. *Results*. Reversal of hyperglycemia after 6 months of PC had no significant effect on nitrotyrosine concentration in the retina, on the nitrotyrosine-positive retinal capillary cells and on the number of acellular capillaries; the values were similar in PC-GC and PC groups. In the same rats retinal MnSOD activity remained inhibited and the total antioxidant capacity was subnormal 6 months after cessation of PC. *Conclusions*. Peroxynitrite accumulation in the retinal microvasculature, the site of histopathology, fails to normalize after reversal of hyperglycemia, and superoxide remains inadequately scavenged. This failure of reversal of peroxynitrite accumulation could be, in part, responsible for the resistance of diabetic retinopathy to reverse after termination of PC.

## 1. INTRODUCTION

The DCCT and the follow-up EDIC studies have shown that instituting tight glycemic control in diabetic patients does not immediately benefit the progression of retinopathy, and the benefits of good control persist beyond the period of good glycemic control [1, 2]. This phenomenon, commonly termed as “metabolic memory”, is stored early in the course of diabetes, and glycemic control initiated prior to the onset of overt pathology has the most profound long-term impact. The persistence or progression of hyperglycemia-induced microvascular alterations during subsequent periods of normal glucose suggests that previous high or low glucose levels imprint their effects, and could result either in resistance of the microvascular complication to halt, or have a long-lasting benefit of good glycemic control [3, 4].

Hyperglycemia, per se, is considered as the initiating factor in the development of retinopathy [5], and various retinal abnormalities induced by hyperglycemia, including elevated polyol pathway activity, increased nonenzymatic glycation, and advanced glycation end products, oxidative stress, and protein kinase C activity [6–9], evidently contribute to the development of the microangiopathy. Superoxide and nitric oxide (NO) levels are elevated in the retina and its capillary cells in diabetes [9–11]. Superoxide anion and NO interact forming peroxynitrite. Peroxynitrite, although not a free radical, can induce lipid and protein oxidation, and nitration of proteins [12]. The expression of nitrotyrosylated proteins is elevated in the retina early in the pathogenesis of diabetic retinopathy, and remains elevated when the pathology is developing [10, 13, 14].

The metabolic memory phenomenon is also observed in animal models of diabetic complications; retinopathy continues to progress for a considerable period even after hyperglycemia is corrected in dogs [15], and islet transplantation after several months of diabetes in rats arrests the progression of retinopathy less effectively than if intervention occurs after only a few weeks of diabetes [16]. We have shown that reinstitution of good glycemic control after 2 months of poor glycemic control in rats inhibits elevations in retinal lipid peroxides and NO levels, but if good glycemic control is reinstituted after 6 months of poor glycemic control, oxidative stress and nitrative stress remain elevated in the retina [17]. Further, reinstitution of normal glycemia after 6 months of poor glycemic control does not inhibit the activation of apoptosis execution enzyme, caspase-3, and the nuclear transcription factor in the retina [18]. However, the exact mechanism of this metabolic phenomenon remains to be elucidated.

The retina is a complex tissue with multiple cell types. In the present study, we have investigated the effect of reestablishment of good glycemic control in diabetic rats on peroxynitrite accumulation in the capillaries of the retina, the major site of histopathology in diabetes, and have compared the nitrotyrosine staining with the appearance of early histological lesions. Effect of reestablishment of good glycemic control was also determined on the activity of the enzyme responsible for scavenging superoxide, one of the major components of peroxynitrite formation.

## 2. METHODS

### 2.1. Animals

Lewis rats (male, 200 g) were assigned to normal or diabetic groups. Diabetes was induced with intraperitoneal injection of streptozotocin (55 mg/kg BW). Diabetic rats were divided at random among 2 groups according to the intended degree of glycemic control. Group number 1 consisted of rats that were allowed to remain in poor glycemic control for 12 months (PC), and group number 2 had rats that were allowed to remain in poor glycemic control for 6 months and this was followed by good glycemic control for 6 additional months (PC-GC). A group of rats remained normal for the entire duration of the experiment. Each group had 10 or more rats.

### 2.2. Glycemia

All diabetic rats received insulin (NPH); the dose and frequency of insulin were adjusted based on the severity of hyperglycemia. The rats in which poor glycemic control was intended received a single injection of insulin (1-2 units) 4-5 times a week to prevent ketosis and weight loss, and the rats in which good control was intended received insulin twice daily (6-8 units total) to maintain their blood glucose levels below 150 mg/dl [18]. The entire rat colony was housed in metabolism cages. Twenty-four-hour urine samples were tested daily for glycosuria with Keto-Diastix (Bayer Corporation, IN), and 2-3 times every week using quantitative methods (Glucose Kit, GAGO-20, Sigma-Aldrich Chemicals, St. Louis, MO). Blood glucose was measured once a week (Glucometer Elite, Bayer Corporation, IN) and glycated hemoglobin (GHb) every 2-3 months using a kit from Helena Laboratories (Beaumont, TX). The rats received powdered diet (PURINA 5001); their food consumption was measured once and body weights 2-3 times every week. These experiments conformed to the ARVO Resolution on Treatment of Animals in Research, as well as to the specific institutional guidelines. At the end of the desired duration of glycemic control, the animals were sacrificed by an overdose of pentobarbital. One eye from each animal was placed into buffered formalin for isolation of the retinal vasculature by the trypsin digest technique (described below), and the other eye was used immediately to isolate retina for biochemical measurements by gently separating sensory retina from choroid using a microspatula under a dissecting microscope.

### 2.3. Retinal microvessels and immunostaining

Retina was removed from the eyes that were fixed in formalin, washed overnight with distilled water, and digested for 90 minutes with 3% crude trypsin in Tris-HCl buffer (pH 7.8) containing 0.2M sodium fluoride to isolate microvessels [14, 18]. These trypsin-digested microvessels were used to localize nitrotyrosine (a biomarker of peroxynitrite formation), and to count the number of acellular capillaries. Microvessels were air-dried on the slides and mounted using 50% glycerol. For nitrotyrosine immunostaining, the preparation was rehydrated with TBS (50 mM Tris-HCl, pH 7.5 and 150 mM NaCl), and incubated with 3% H_2_O_2_ for 15 minutes, followed by rinsing with TBS to remove excess H_2_O_2_. The samples were blocked with 5% goat serum for 1 hour at room temperature followed by incubation with antinitrotyrosine antibody (Upstate, Lake Placid, NY). The slides were washed 3 × 10 minutes with TBS, and incubated with peroxidase-conjugated secondary antibody (goat antirabbit) for 1 hour at room temperature. The microvessels were washed 3 × 10 minutes with TBS, and stained with aminoethylcarbazole solution (in 0.1 M acetate buffer, pH 5.2, containing 0.05% H_2_O_2_) for 40 minutes. The slides were counterstained with Gill's Hematoxylin solution for 2 minutes, rinsed with TBS, and visualized under a microscope.

The numbers of nitrotyrosine positive cells and acellular capillaries were counted in multiple midretinal fields (one field adjacent to each of the 5 to 7 retinal arterioles radiating out from the optic disc) and expressed per mm^2^ of retinal area examined [9, 14].

### 2.4. Nitrotyrosine concentration in the retina

Nitrotyrosine concentration was quantified by enzyme immunoassay using a Nitrotyrosine-EIA kit from Oxis Research (Portland, OR) as described in [14, 19]. Nitrotyrosine standard or retinal homogenates were incubated with nitrotyrosine antibody in the microplate for one hour, and antinitrotyrosine antibody for one hour; this was followed by incubation with streptavidin peroxidase for one hour. The samples were incubated with tetramethylbenzidine substrate for 30 minutes, and the reaction was stopped by 2.0 M citric acid. The formation of yellow product was measured at 450 nm. The assay was sensitive to concentrations as low as 0.05 pmoles of nitrotyrosine.

### 2.5. Enzyme activity of MnSOD

The activity of SOD was measured in the retina using 5–10 *μ*g protein by a method that utilizes tetrazolium salt to quantify superoxide radicals generated by xanthine oxidase and hypoxanthine. MnSOD activity was determined by performing the assay in the presence of potassium cyanide to inhibit Cu-Zn SOD, and the residual MnSOD activity was calculated [19].

### 2.6. Total antioxidant capacity

The total antioxidant capacity of the retina was measured using a kit from Cayman Chemical (Ann Arbor, MI) as described in [19]. The assay is based on the ability of the sample to inhibit oxidation of 2,2'-azino-di-[3-ethylbenzthiazoline sulfonate] ^R^ (ABTS) by metmyoglobin; the antioxidants in the sample cause decrease in absorbance at 750 nm, and that represents the amount of ABTS^R+^ produced. Each measurement was made in duplicate using about 5 *μ*g of retinal protein.

### 2.7. Statistical analysis

The results are reported as mean ± SD and analyzed statistically using the nonparametric Kruskal-Wallis test followed by Mann-Whitney test for multiple group comparisons. Similar conclusions were reached also by using ANOVA with Fisher or Tukey.

## 3. RESULTS

### 3.1. Glycemia

The rats in PC group, as expected, had severe hyperglycemia; their GHb values were about 12% throughout the 12 months duration compared to less than 6% in the rats that remained normal for the entire duration of the experiment (Table 1). The GHb values in the rats in PC-GC group were around 12% before good glycemic control was initiated, but decreased by 2 fold after reinstitution of good glycemic control in those rats.

In the same PC-GC rats, body weight and daily food consumption before reinstitution of good glycemic control were statistically similar to those obtained from the rats in PC group, but became comparable to the normal rats after reestablishment of good glycemic control (Table 1). Similar pattern was observed for 24-hour urine excretion (data not shown).

### 3.2. Nitrotyrosine and histopathology

**Figure 1:**
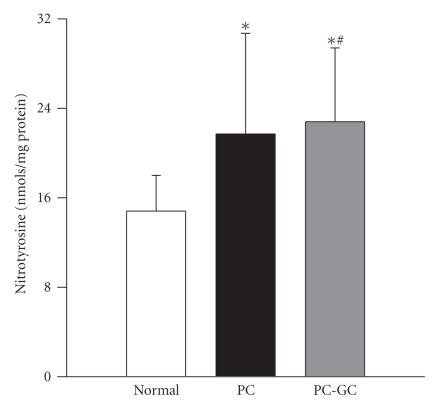
Effect of cessation of hyperglycemia on nitrotyrosine levels in the retina. Nitrotyrosine was quantified in the retinal homogenate using a Nitrotyrosine-EIA kit. Each sample was measured in duplicate. The figure represents mean for all SD of 7 rats in normal, 6 rats in PC, and 8 rats in PC-GC groups. *P < .05 compared to normal, and ^#^ *P* > .05 compared to PC.

The total nitrotyrosine concentration in the retina was elevated by about 30% in the rats that were maintained in poor glycemic control for the entire duration of the experiment (12 months) compared to the age-matched normal rats (Figure 1). Reestablishment of good glycemic control after 6 months of poor glycemic control failed to decrease nitrotyrosine levels in the retina; both PC and PC-GC groups had similar concentrations, and these values were significantly different from those obtained from the normal control rats (*P* < .05).

**Figure 2:**
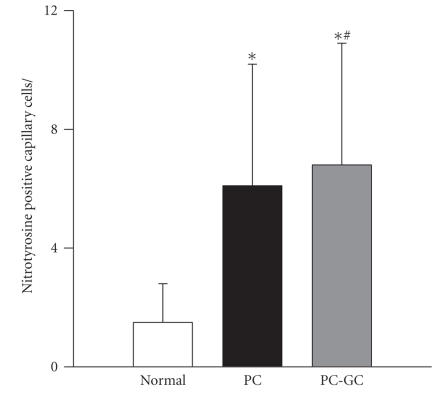
Effect of reversal of hyperglycemia on nitrotyrosine-positive capillary cells in the retina. Trypsin-digested retinal microvessels were immuno-reacted with antinitrityrosine antibody, and stained with aminoethylcarbazole solution 0.05% H_2_O_2_ for 40 minutes. This was followed by counterstaining with Gill's Hematoxylin solution. Nitrotyrosine stained capillary cells were counted in a masked fashion. The results are obtained from 7–9 rats in each of the 3 groups. *P < .05 compared to normal, and ^#^ *P* > .05 compared to PC.

In order to determine nitrotyrosine levels in the retinal microvasculature, trypsin-digested microvessels were stained with the antibodies that detect nitrotyrosine. The number of nitrotyrosine positive cells was about 2.5 fold higher in the microvessels prepared from the retina of the rats in PC group, compared to the rats that remained normal for the entire duration of the experiment. Six months of poor control followed by 6 additional months of good control (PC-GC group) did not produce any beneficial effect on the number of nitrotyrosine positive capillary cells; the values were similar in the microvessel preparations from the PC and PC-GC groups (Figure 2).

**Figure 3:**
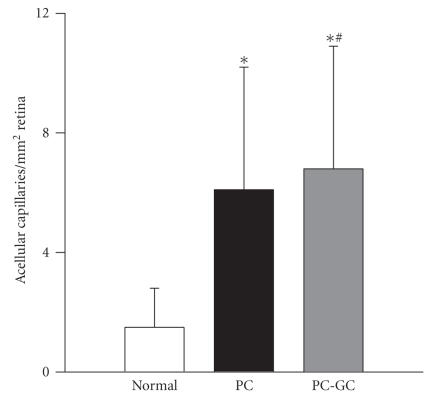
Reversal of hyperglycemia and retinal histopathology. The number of acellular capillaries was counted in multiple midretinal fields in a blinded manner in the trypsin-digested retinal microvessels that were used for nitrotyrosine staining. *P < .05 compared to normal, and ^#^ *P* > .05 compared to PC.

The histopathology associated with diabetic retinopathy was evaluated in the same trypsin-digested retinal microvessel preparation. Figure 3 shows that the number of acellular capillaries was increased by about 4 fold in the retina of rats in PC group compared to the age-matched normal rats. Reinstitution of good glycemic control (PC-GC) failed to provide any significant effect on the number of acellular capillaries in the retinal vasculature; the number of acellular capillaries remained significantly elevated in the PC-GC group compared to the normal group.

### 3.3. Superoxide dismutase activity

**Figure 4:**
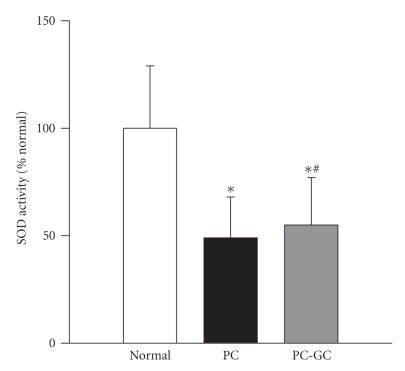
Effect of reversal of hyperglycemia on MnSOD enzyme activity in the retina. Retinal protein (5–10 *μ*g) was used to measure SOD activity using an assay kit (Cayman Chemical, Ann Arbor, MI). MnSOD activity was calculated by subtracting the potassium cyanide-inhabitable activity from the total SOD activity, and the activity obtained from the retina of normal rats was considered as 100%. The values are mean ± SD of 7-8 rats in each group. *P < .05 compared to normal, and ^#^ *P* > .05 compared to PC.

The enzyme activity of MnSOD was inhibited by 50% in the retina of rats diabetic for 12 months (PC group) compared to the age-matched normal rats (Figure 4). Six months of good glycemic control that followed poor glycemic control did not reverse the inhibition of MnSOD activity; the enzyme activity in PC and PC-GC groups was not different from each other (*P* > .05).

### 3.4. Total antioxidant capacity

**Figure 5:**
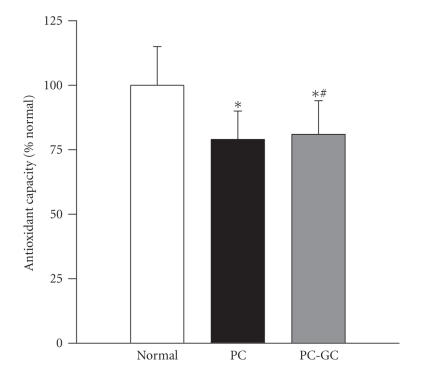
Effect of reversal of hyperglycemia on the total antioxidant capacity of the retina. The total antioxidant capacity was measured in the retina (5–10 *μ*g) using a kit from Cayman Chemical, MIBTS. Each sample was measured in duplicate, and the values are represented as mean ± SD of 8 normal, 7 PC, and 9 PC-GC rats. *P < .05 compared to normal, and ^#^ *P* > .05 compared to PC.

The overall antioxidant capacity of the retina, as expected, decreased by about 25% in the rats that were in the PC group compared to their age-matched normal rats. Reinstitution of good glycemic control after 6 months of poor glycemic control had no beneficial effects on the diabetes-induced decrease in the total antioxidant capacity of the retina; the values obtained from the rats in PC-GC group remained significantly lower than the normal rats, and were not different from those obtained in the PC group (Figure 5). 

## 4. DISCUSSION

This is the first report demonstrating that peroxynitrite accumulation in the capillaries of the retina, the site of histopathology in the development of diabetic retinopathy, resists arrest after reinstitution of good glycemic control in the rats that has followed a period of poor glycemic control. In the same rats, reversal of hyperglycemia fails to inhibit the development of retinal histopathology. This strongly suggests that diabetes-induced nitrative modifications in the capillaries of the retina play an important role in the metabolic memory phenomenon. These novel findings from the retinal capillaries are supported by our previous data obtained from the whole retina demonstrating that retinal oxidative stress and expression of nitrosylated proteins remain elevated 7 months after reinstitution of good glycemic control in the rats that had 6 months of poor glycemic control [17, 18], and also from experimentally galactosemic rats, another animal model of diabetic retinopathy, showing that the expression of retinal nitrosylated protein is elevated for at least 1 month of galactose-withdrawal that has followed 2 months of 30% galactose diet [20]. Further, we also provide evidence that the reversal of hyperglycemia has no beneficial effects on the inhibition of the enzyme responsible for scavenging mitochondrial superoxide, MnSOD, and on the overall antioxidant capacity of the retina.

Our data show that reversal of poor glycemic control by reinstituting good glycemic control with insulin regimen had no significant effect on the retinal histopathology in rats. In support, others have shown that islet transplantation, if performed 12 weeks after induction of diabetes in rats does not prevent retinal vessel occlusion [16], and reinstitution of good glycemic control for 2.5 years in dogs after 2.5 years of poor control does not benefit the development of diabetic retinopathy [15]. In experimentally galactosemic rats and dogs, withdrawal of galactose after a period of galactose-diet does not completely prevent the progression of retinal pathology [21, 22]. Our exciting results show that in the same retinal microvascular preparation the number of nitrotyrosine-positive capillary cells does not decline 6 months after good glycemic control is reinstituted in diabetic rats. This is the first report suggesting that peroxynitrite accumulation in retinal capillaries could be contributing to the failure of retinopathy to reverse.

Peroxynitrite formed by the reaction between superoxide and NO can attack a wide range of biological targets, including proteins and DNA [12]. Peroxynitrite nitrates free tyrosine and tyrosine residues in proteins [23]. Nitration of proteins can disrupt protein assembly and functions with possible pathological consequences [24], and is postulated to be involved in the apoptosis of retinal cells [25]. Increased peroxynitrite is also associated with various chronic diseases, including Parkinson's disease, Alzheimer's disease, and diabetes [26, 27]. In diabetes the levels of superoxide and NO are elevated in the retina [9–11], and the expression of nitrosylated proteins and the concentration of nitrotyrosine are increased [10, 13, 14]. Further, reinstitution of good glycemic control does not easily reverse diabetes-induced increase in the protein expression of nitrosylated proteins in the retina [17]. We demonstrate that diabetes-induced increase in the nitrotyrosine positive capillary cells, and also the total retinal nitrotyrosine concentration resist reversal after reestablishment of good glycemic control, suggesting that peroxynitrite, which starts accumulating in the retinal capillaries during poor glycemic conditions, once formed, becomes difficult to reverse. Nitrotyrosine in the retina and its capillary cells was measured at 12 months duration, however, our previous results have shown that the duration of diabetes and the age of the rat (2–14 months) have no significant effect on the increase in retinal oxidative stress and nitrative stress [9, 13, 17, 18]. In addition, although there is no clearly defined mechanism of removal of nitration, there are some reports showing that the enzyme denitrase can remove the nitro group without degrading the protein [28]. Our results showing the failure of reversal of nitrotyrosine imply that the activity of this enzyme could be affected by sustained high glucose, and is not easily normalized after removal of high glucose.

Mitochondria are considered as the major source of superoxide production and are subjected to direct attack by reactive oxygen species (ROS) [29]. Superoxide act as a causal link between elevated glucose and the major vascular complications in diabetes [3]. The levels of superoxide are elevated in the retina in diabetes, cytochrome c release from the mitochondria to the cytosol is increased, and the mitochondrial electron transport chain is impaired [11, 19, 30, 31]. MnSOD is the first line of defense; it scavenges superoxide anion in the mitochondrial matrix by catalyzing dismutation of superoxide radicals via conversion of superoxide to hydrogen peroxide and oxygen [32]. MnSOD protects the disruption of mitochondrial membrane potential, and its inhibition results in increased superoxide radicals [33]. The activity of MnSOD is decreased in the retina in diabetes, its expression is down regulated, and the therapy that inhibits the development of retinopathy in diabetes also inhibits diabetes-induced decrease in MnSOD activity [19, 31]. Overexpression of MnSOD protects the retina from diabetes-induced decrease in antioxidant capacity and GSH levels, and increase in the oxidatively modified DNA levels implying that MnSOD could be important in protecting the retina from diabetes-induced damages [19]. Here we provide data to show that diabetes-induced inhibition of retinal MnSOD activity does not reverse 6 months after good glycemic control is reinstituted. This suggests that superoxide that were accumulating in excess due to hyperglycemia, continue to be scavenged inadequately, thus contributing to elevated peroxynitrite levels in the retina and its microvasculature. The reason for this failure of MnSOD inhibition to reverse could include inactivation of the active site of MnSOD via either increased nonenzymatic glycation or nitration, or any other post-translational modification that would be difficult to reverse [34, 35]. Our results of increased nitrotyrosine levels in the retina of PC-GC rats suggest that MnSOD could remain nitrosylated compromising the scavenging of superoxide, and contribute to the persistent increased oxidative stress even after reinstitution of good glycemic control. Diabetes-induced decrease in the retinal antioxidant capacity, a measure of the total protective antioxidant mechanism [36], was not normalized after reversal of hyperglycemia in rats, suggesting that the failure could be either due to continued increased production of free radicals, or their decreased removal, or both. We have shown that retinal antioxidant capacity is decreased in the retina in diabetes, and MnSOD protects this diabetes-induced decrease [19]. Thus, the failure of reversal of inhibition of MnSOD activity after reestablishment of good control could account, in part, to the decreased total antioxidant capacity of the retina. Further, this resistance of the total antioxidant capacity of the retina to become normal could be due to sustained decreased levels of intracellular antioxidant, GSH. In support, we have shown that the reinstitution of good glycemic control in diabetic rats does not produce beneficial effects in increasing the concentration of retinal GSH levels [17].

Thus, data presented here strongly suggest that peroxynitrite accumulation in the microvasculature of the retina and impaired scavenging of mitochondrial superoxide could be important elements in the failure to halt the progression of diabetic retinopathy. Identifying the abnormalities responsible for the resistance of retinopathy to reverse after establishment of normal blood sugar levels should reveal novel targets for therapies to prevent the progression of retinopathy in diabetic patients.

## Figures and Tables

**Table 1:** Reinstitution of good glycemic control and the severity of hyperglycemia. The rats were weighed 2 times a week, and the food consumption was measured once/week. Glycated hemoglobin was measured every 2 months using a kit from Helena Laboratories. The values are mean ± SD of 7 rats in normal group and 8 rats each in PC and PC-GC groups. *P < .05 and ^#^ *P* > .05 compared to normal.

	Body weight (g)	Food (g/day)	Glycated Hemoglobin(%)

Normal	384 ± 26	23 ± 3	6.7 ± 0.6
Poor control	281 ± 34*	37 ± 6*	13.3 ± 2.2*
Poor control	265 ± 25*	41 ± 9*	14.7 ± 2.1*
↓	↓	↓	↓
Good control	334 ± 36^#^	25 ± 5^#^	6.9 ± 1.1^#^
